# Extracts

**Published:** 1883-05

**Authors:** 


### Extracts.

---

Intra-Uterine Injections in the Treatment of Puerperal Septicæmia.—The following case seems to me to illustrate what should be the accepted treatment of puerperal fever, or puerperal septicæmia, at the present day. The case was that of a lady in the higher walks of life whom I was called to see about a month ago, in consultation by her physician, a man of wide experience. She was a primipara, was taken in labor at four o'clock Sunday afternoon, and at nine o'clock in the evening was delivered of a female child, without any difficulty or assistance. Her physician examined the external genitalia carefully, and found no tear whatever. The nurse was instructed to syringe out the vagina carefully the next day with carbolized water, which she did. The first forty-eight hours passed by without any bad symptoms at all, but, on visiting her on Tuesday morning, the physician found a temperature of 101° F., and in the evening it had risen to 102·5°. The next morning, the morning of the fourth day, the temperature was 103°, and the patient began to complain of very severe pain in the right iliac fossa. There had been no chill. At five o'clock in the afternoon the tempereature was 106·° in the mouth. The patient's appearance became wild, as of one who was about to have puerperal mania; the skin was hot, and she was crying out with pain, although she had received a good deal of morphine.

Having now been called to see the patient, I took the temperature in the mouth myself, and confirmed the record of her physician, that it was 106·5°. The pulse was 145. Making a vaginal examination, I found a bilateral laceration of the cervix uteri extending nearly up to the vaginal junction. Probably this extensive laceration partly accounted for the rapidity of the labor as occurring in a primipara. I urged that the uterus should be washed out with carbolized water at once, but her physician had never seen the method practiced, and was strongly prejudiced against it; he finally consented only because it was apparent that unless something decided was done the patient would soon die. Using the Chamberlain tube and the Davidson syringe, Dr. Jones, and afterward Dr. McCosh, continued to wash out the uterus with carbolized water every four hours during the night, and the next morning the temperature was found to have sunk from 106 5° to 101°; the pulse had fallen from 145 to 120; the patient, who had ueen given opium quite freely during the night, declared that she was very much relieved, Indeed, the relief had been so extraordinary that they began to believe that the danger was not real at all; that some exceptional circumstance had occurred, and that there was no septicæmia. The uterus was now washed out at longer intervals, but at once the temperature went up to 102°, 103°, 104°, and 105°, and the patient again began to look manical. The uterus was now washed out every three hours, opium was freely administered, ten grains of quinine were administered every eight hours, ice-water was passed through a coil of rubber tubing placed over the abdomen; and as long as this treatment was kept up the temperature did not rise above 101° or 100°; but so soon as they ceased to wash out the uterus the temperature at once rose to 104°, and at times to 105°. This fact was proved by repeated trials.

After this treatment had been continued for ten days, a physician remaining with the patient day and night, giving the injections every three hours, and thirty grains of quinine during the course of the day, it was believed to be time to stop it; but in less than twenty-four hours the temperature again rose to 105°. I mention the amount of quinine which was being taken particularly, so as to prove positively that there was nothing of a malarial character in the case at all. On the sixteenth day after delivery, the tenth day after the commencement of the high temperature, the intervals between the uterine injections were extended from three hours to four, then to five, six, and seven hours, and finally they were discontinued altogether, and at the same time the administration of quinine was given up and the coiled tubing was taken off. Opium was continued in small doses for a while longer, and the patient recovered entirely.

I wish to contrast this case with another which I saw just before—that of a woman who had been recently delivered of her third child. When I was called to see the patient the temperature was 106°; she had been taken with violent pain in one iliac fossa, and had been put five days before pretty profoundly under the influence of opium, and a blister had been applied over the whole of the abdomen. Large doses of quinine had likewise been administered. When I saw the patient the use of intra-uterine injections was begun at once, but the patient lived only twenty-four hours, and died in a state of coma.

It seems to me that the time has arrived when puerperal septicæmia should be treated upon just as simple a plan as septicæmia of any other kind is, namely, by washing with some antiseptic fluid the surface where the disease originates—some fluid which will remove the poisonous material which is being absorbed, and also, so far as possible, neutralize its poisonous qualities. In brief, I would say that puerperal septicæmia, with our present light on the subject, should be treated in the following manner: First, wash out the uterine cavity completely with some antiseptic fluid; second, quiet all pain by opium; third, get the peculiar influence of quinine upon the nervous system; and, fourth, keep the temperature, at all hazards, at or below 100° by the methods which we now possess. Three years ago, at the American Gynæcological Society, which met in Baltimore, I took the ground which I take to-day regarding this subject, and only one gentleman in the entire society supported my view. Every other member who spoke referred to the dangers of introducing air into the uterine sinuses during the injection etc. But I believe that the dangers attending the use of the injections are counterbalanced by the benefits to be derived. I do not think there is the least probability that air will be introduced if a tube of large size—as large as the finger—is used. But when a catheter is employed there is some danger of inserting it into a sinus and introducing air and fluid together directly into the vessels.—*T. Gaillard Thomas, M.D, in N. Y. Med. Jour.*

---

The Treatment of Diphtheria.—In the *Southern Clinic* for January, 1883, Dr. A. C. Fox desires to call the attention of the profession to the early engorgement of the deep cervical glands, which when once seen and felt, can never be forgotten. He thinks when this symptom is once appreciated, there will be fewer deaths from diphtheria. The importance of the symptom he learned to appreciate nearly twelve years ago.

"I will give you my treatment for a child two years old. The dose can be increased or diminished according to age.

"When first called, I prescribe:

℞ Hyd. chlor. mit........................gr. vj.
Precip. chalk........................gr. xv.
Pulv. rhei............................gr. ij.

Mix and divide into six powders.

S.—One every four hours until they act well on the bowels, and follow with castor oil in four or five hours if necessary.

If the bowels are too lax, leave off the rheum, and add Dover's powders. At the same time I prescribe:

℞ Sulph, quinine......................gr. xvj.

Pulv. chlorate potassa...............ʒj.

Mur. tr. iron.........................ʒss.

Water.................................℥ij.

Dissolve the potass. in the water, and add the other ingredients.

S.—Teaspoonful every two hours day and night until there is a decided improvement, then every three hours.

If the case improves I give it three times a day until the cervical glands have become natural in size. If the fever returns, I instruct the nurse to give the solution as at first, and inform me.

I always use the following liniment to establish counter-irritation as soon as possible. There is no danger of establishing too much. I invite the disease outside where

I can see it, and then I feel that the larynx and trachea are safe.

R Croton oil ............................... ʒj.

  Alcohol.................................... ʒj.

M. S.—Apply twice a day until the skin gets thoroughly red.

Put a flannel cloth around the neck next to the skin until the skin gets sore, then put a linen between.

I seldom fail to find my patient much improved in twenty-four or thirty-six hours, when called early.

If the case proves obstinate, I give another round of calomel in two or three days. I always see that the bowels move once or twice well in twenty-four hours.

I give plenty of hot milk, animal broths or soup, eggs and egg-nog, and a teaspoonful of whisky every two or three hours from first to last.

If the patient is old enough to gargle, I let him use chlorate pot., a strong solution, or a weak red-pepper tea with salt in it, or a little carbolic acid in water. I have no confidence in internal local applications. In the croupous form, lime inhalations with the above treatment is all I ever find necessary.

If the patient has no appetite, I give food as medicine.

---

ON THE DETECTION OF THE BACILLI OF TUBERCLE IN THE BREATH OF CONSUMPTIVE PATIENTS.—Dr. R. Charnley Smith (*Brit. Med. Jour.*, January 20, 1883,) says: By the following simple method, I have succeeded in demonstrating with facility the presence of the baccilli of tubercle in the breath of patients suffering from true tubercular consumption; for which purpose I allow the patient to breathe, at frequent intervals during the day, through two thin sheets of pyroxylin, or fine cotton, one layer in front of the other, and both of which are placed in the outer compartment of an ordinary "pepper-duster" respirator. The layer of cotton, when so arranged, will act as a double filter, the external layer removing from the ingoing air all suspended particles, such as dust, micro-fungi, polur, starch, etc., which are always more or less present in it, and which it is desirable to exclude; that portion of cotton which has been next to the mouth at the same time retaining only those existing in the outgoing current, and which have been emitted from the lungs, viz., micrococci, bacilli, and some epithelial cells. It is in the latter layer only that I look for the organisms peculiar to this disease. This I do by converting the pyroxyline into gun-collodion by means of a mixture of ether and spirit. Every vestige of cotton-fibre is dissolved in the above menstruum, but other organic particles remain suspended in it. To render the bacilli manifest, my plan is to pour the thin collodion thus formed on a microscope-slide, and allow the fluid to run uniformly over the surface of the glass, then immediately placing the latter on one of its edges, that only the merest film of collodion may remain on the glass; the thinner the film produced, the more successful will be the experiment. The film is to be stained. This may be done by one of the methods well known to the profession for staining tuberculous sputum, such as that of Ehrlich or Heneage Gibbes.

---

ADULTERATION OF BISMUTH SALTS.—The *Ephemeris of Mat., Med. etc.*, says: A sample of so-called submitrates of bismuth was recently sent for examination, having been sold in a paper parcel by a wholesale drug house of New York as the product of a respectable maker, and with the maker's name on the label.

From the depth of the yellow tinge it was evidently not a proper subnitrate, and was supposed to be subcarbonate; but as it did not effervesce with an acid, it was not a subcarbonate. It contained a number of very small hard lumps, which, when broken or rubbed to powder, were lighter in color than the pulverulent portions. Both powder and lumps had a very decidedly saline taste, but the lumps were more saline than the powder, and they seemed to melt on the tongue. The whole, when well washed with distilled water, gave a solution which was not affected by hydric sulphide (sulphuretted hydrogen), and therefore no bismuth. But it should have contained bismuth had there been any nitrate or other soluble salt of bismuth present. A weighed portion of the washings evaporated to dryness left a residue amounting to about 30 per cent. of the weight of the original sample. This residue was neutral, and contained nitric acid and soda, and was probably nearly all nitrate of soda. The sample, therefore, consisted approximately of say 70 per cent. oxide of bismuth, and 30 per cent. nitrate of sodium, without any subnitrate of bismuth at all, although sold under that name. It seems to have been made by precipitating a solution of nitrate of bismuth by a solution of soda, and by separating and drying the precipitate with little or no washing.

Now, this was ordered in paper as the subnitrate of bismuth of a specified manufacturer, and was sold by one or two wholesale druggists who attract much trade by low prices, but who do not themselves manufacture anything, so far as is known. Such, however, in order to sell at low prices, must buy at correspondingly low prices to preserve their profits. It is probable, therefore, that such a product as this was bought as a "job lot" from some irresponsible seller who made it, or had it made, as a bad counterfeit of the manufacturer whose name it bore ; and if bought cheaply enough it was not to the interest of the wholesale house who bought it to inquire too closely as to its genuineness or its true character.

The dispensing pharmacist who buys and dispenses such articles shares about equally in the criminality with the wholesale house and the maker, because he goes to such houses voluntarily for the sake of buying cheaply. He buys in paper parcels for the sake of avoiding the cost of a bottle, which is not only necessary for the proper preservation of most medicines, but which aids materially in identifying the product of any maker. And finally, he dispenses such products without exercising the knowledge which he should, and generally does possess, of discriminating between a good preparation and one which is grossly adulterated—in this case so grossly that a mere casual inspection was sufficient to detect the fraud.

Many pharmacists are led into such purchases as this by insisting upon having their preparations put up in paper parcels. They have shop bottles for their preparations, and cannot see the necessity of buying a bottle each time they require a new supply. Therefore they will buy of those houses and of those manufacturers only who will put their wares up in paper. No known manufacturer of bismuth salts willingly sends out his product in paper, but all do it under a kind of tacit protest under the pressure of the pharmacist and physician, and thus the remote consumer, namely, the patient, suffers in consequence.

It is true that the bottle and label do not guarantee the contents, because all manufacturers know that their labeled empty bottles are often refilled with products for which they are in no degree responsible. Yet, as this fraudulent buying up and refilling of labeled bottles is much more difficult, and involves much more risk, it is less frequently done. Consequently, there can be no doubt that if pharmacists and physicians would refuse to take, or would even cease to demand, important medicines in paper parcels, they would be much less frequently deceived in the character of their supplies, and they would thus admit, what they ought to know better than any manufacturer, that all medicines which are made with due care and accuracy are well worth the containing vessel necessary to keep them in their original condition as made.

It is a common fallacy that inexpensive articles should always be put in paper, and hence alum, muriate of ammonia, chlorate of potassa, sulphate of zinc, etc., are very commonly ordered in paper, whereas they are in reality of as much value as medicines as are the bismuth salts and other more costly articles; the circumstance that the bottle costs half as much as the contents being merely incidental.

The habit of ordering important medicines in paper is rapidly increasing; and as it increases with physicians as well as pharmacists, it is fair to infer that the mistake is upon both, while the blame is greatest upon the physician, who is most directly responsible in the application of medicines. And if the physician would but use his influence earnestly against it, the practice would soon cease altogether. If he will not do this, there will then be need for some manufacturer who will lose the sale of his medicines rather than put them up in paper.

No reasonable amount of care and expense is misapplied to medicines, and it is very disheartening to a manufactu- ıel to see all his care, pains and pride in his productions risked by putting them up in paper because pharmacists and physicians will not pay for proper containing vessels, but will send back articles and buy elsewhere if their orders for paper percels are disregarded.

---

The Therapeutic Use of Clay.—The London *Medical Record*, February 15, 1883, says that Dr. M. V. Saveljeff, of Vladimir, confirms (*Vratch*, 1882, No. 16) the results published by Drs. D. T. Sokoloff (*London Medical Record*, April 1882, p. 144) and A. Masalitanoff (*Ibid.*, Nov., p. 452). Having followed the advice of Professor Botkin, he has since 1878 employed clay-cakes in about one hundred cases of cardiac organic diseases, cardiac neuroses, and palpitations of every kind, and invariably met with most remarkable success. He takes common red clay (instead of Sokoloff's sculptor's clay, or Masalitanoff's plaster of Paris), moistens it with water until a thick paste is formed, makes a cake of a palm's size and of a finger's thickness, and then applies it to the cardiac region. Pain disappears within eight or ten minutes, palpitation within ten to fifteen. A cake dries in feverish patients in two or three hours, otherwise in five, and then is to be changed. Clay-cakes are beneficial not only in cardiac cases. The author himself has suffered from paroxysmal neuralgia in the region of the left kidney, the attacks being very severe, and lasting from six to eight hours, in spite of the use of narcotics. Of late, he began to apply clay-cakes to the painful region every time when a paroxysm was coming. Since then, the paroxysms have had a duration of only half an hour or so, and appear far more rarely. In conclusion, the author states that the Vladimir peasants from time immemorial have employed red clay-cakes (all over the body) as a cooling remedy in febrile cases of every description.

Baking Soda for Burns.—The *California Medical Journal* says: Some of our readers may not know the benefit to be derived from common baking soda, in treating burns. We learned this somewhere a number of years ago, and have frequently verified in practice what we have often seen reiterated in the prints, that this is the remedy *par excellence.* It should be used freely. A good way is to moisten the powdered bi-carbonate until it may be spread, like paste, upon muslin, when it should be closely adjusted to the injured surface. It will often relieve the pain like magic. A striking illustration of this came to our notice a few weeks ago. A lad, ransacking a blacksmith shop, burned his hand severely upon a horseshoe, just long enough out of the fire to lose its red heat. He ran home in agony, and continued to suffer intense pain for several hours. About nine o'clock in the evening the soda was applied, and in a short time he was lost to all discomfort in a tranquil sleep. No pain or unpleasantness was experienced in the affected part afterwards, and it healed rapidly.

---

A Note on the Sulpho-Carbolates.—Dr. H. P. Farnham, of New York, read a paper on the Sulpho-Carbolates of soda and zinc, which he strongly advocates in the earlier stages of diphtheria, before the Materia Medica Society (*Med. Record*, January 6, 1883). When well prepared, he considers them invaluable; when not pure they are disagreeable and unsatisfactory. The danger is contamination with: 1, the sulphate of the base employed; 2, the carbolate, which evolves the odor of carbolic acid; 3, free sulphuric acid; 4, free carbolic acid. The best method is to produce, first, a definite sulpho-carbolic acid by taking equivalent weights of its components, and to neutralize with the calculated weights of the oxides employed. Crystalization should take place slowly without heat.

*Tests.*—Each sulpho-carbolate should possess a definite and decided crystalline form. They should give off scarcely any odor of carbolic acid; they should give no precipitate with barium chloride.

